# An audit on consent- risk disclosure in thoracic surgery

**DOI:** 10.1186/1749-8090-10-S1-A204

**Published:** 2015-12-16

**Authors:** Sparsh Prasher, Laura Castle, Michael Klimatsidas, Zahid Mahmood

**Affiliations:** 1 Golden Jubilee National Hospital, Agamemnon St, Clydebank, Dunbartonshire G81 4DY, UK

## Background/Introduction

The law on consent is continuously evolving as there is no statue in British law relating to consent. Instead this relies on common or case law. The recent case of Montgomery vs NHS Lanarkshire has change the consent law with relation to the disclosure of information. It is now recommended that we as surgeons should disclose risks that an average patient would want to know. In modern surgical practice consent should be viewed as a continuous process involving an honest and open discussion and should take into consideration the patient's rights, values and choices.

## Aims/Objectives

To assess the information given to patients during the consent process for thoracic surgery.

## Method

We retrospectively examined the clinic letters, consent forms and case notes for patients undergoing surgery in the thoracic department over a 2 week period in May 2015.

## Results

Over a Two-week period 21 patients underwent thoracic surgery. Of the 21 clinic letters, only 11 (52%) stated the benefits and risks of the procedure that were discussed with the patient. Only 3 consent forms stated the risks of the procedure. 9 (42%) of case notes had no evidence of any discussion of benefits and risks of the procedure on the clinic letter, case notes or consent form.

A graph is attached which demonstrates the specific risks that were discussed with the patients.

## Discussion/Conclusion

The recommendations on risk disclosure have evolved by moving away from the paradigm of paternalism to emphasize the significance of patient autonomy. It is therefore, important to document the discussion with the patient in the clinic letters as well as consent forms, to prevent litigation in the current legal climate. This audit demonstrates that 42% case notes and consent forms do not have any written documentation of risk disclosure. We have designed a new consent form that lists important risk factors that all patients undergoing thoracic surgery should be informed about. This new consent form will standardise the documentation of the consenting process.

